# MicroRNA-302/367 Cluster Impacts Host Antimicrobial Defense via Regulation of Mitophagic Response Against *Pseudomonas aeruginosa* Infection

**DOI:** 10.3389/fimmu.2020.569173

**Published:** 2020-10-07

**Authors:** Ting Huang, Qinqin Pu, Chuanmin Zhou, Ping Lin, Pan Gao, Xiuyue Zhang, Yiwen Chu, Bisong Yue, Min Wu

**Affiliations:** ^1^ Antibiotics Research and Re-evaluation Key Laboratory of Sichuan Province, Sichuan Industrial Institute of Antibiotics, Chengdu University, Chengdu, China; ^2^ Key Laboratory of Bio-resources and Eco-environment (Ministry of Education), College of Life Sciences, Sichuan University, Chengdu, China; ^3^ Department of Biomedical Sciences, School of Medicine and Health Sciences, University of North Dakota, Grand Forks, ND, United States; ^4^ State Key Laboratory of Biotherapy, West China Hospital, Sichuan University, Chengdu, China

**Keywords:** *Pseudomonas aeruginosa*, miR-302/367 cluster, bacterial elimination, mitophagy, reactive oxygen species

## Abstract

Mitophagy has recently been implicated in bacterial infection but the underlying mechanism remains largely unknown. Here, we uncover a role of microRNA-302/367 cluster in regulating mitophagy and its associated host response against bacterial infection. We demonstrate that miR-302/367 cluster expression was significantly increased after *Pseudomonas aeruginosa* infection. Enhanced expression of miR-302/367 cluster accelerated the mitophagic response in macrophages, thus increasing clearance of invading *P. aeruginosa* by regulating production of reactive oxygen species (ROS), while application of miR-302/367 cluster inhibitors decreased bacterial clearance. Blocking mitophagy with siRNA against mitophagy receptor prohibitin 2 (PHB2) reduced the effect of miR-302/367 cluster on induction of mitophagy and its-associated *P. aeruginosa* elimination. In addition, we found that miR-302/367 cluster also increased bacterial clearance in mouse model. Mechanistically, we illustrate that miR-302/367 cluster binds to the 3′-untranslated region of nuclear factor kappa B (NF-κB), a negative regulator of mitophagy, accelerated the process of mitophagy by inhibiting NF-κB. Furthermore, inhibition of NF-κB in macrophages attenuated the ROS or cytokines production and may reduce cell injury by *P. aeruginosa* infection to maintain cellular homeostasis. Collectively, our findings elucidate that miR-302/367 cluster functions as potent regulators in mitophagy-mediated *P. aeruginosa* elimination and pinpoint an unexpected functional link between miRNAs and mitophagy.

## Introduction

To date, lung disease is becoming the most dangerous disease beyond the HIV/AIDS, cancer and heart disease (1). *Pseudomonas aeruginosa* is a gram-negative, opportunistic pathogen that causes severe respiratory infections, but also compounds situations in cystic fibrosis patients, immunocompromised individuals, and burn patients (2). Once acquired and colonized in the respiratory tract, this bacterium is extremely difficult to eradicate. Although the recognition of pathogen-associated molecular patterns (PAMP) by an infected host has been demonstrated (3), the detailed mechanisms by which innate immune cells responding the invasive pathogen remains incompletely characterized.

Mitophagy, the selective autophagy to eliminate damaged mitochondria, is a highly conserved cellular self-digestion and catabolism process critical for maintaining cellular homeostasis (4). The dysregulation of mitophagy appears to be responsible for many pathogenic infections, indicating the importance of its precise control (5–7). Numerous autophagy-related genes (Atgs) and mitochondrial proteins are critical for activation of mitophagy and phagolysosomal maturation during microbial invasion (5, 8, 9). Additionally, host mitochondria also play key roles in resistance against bacterial infection, such as the regulation of bactericidal reactive oxygen species (ROS) (10) and inflammasome activation (11). Besides Atgs, nuclear factor kappa B (NF-κB), a ubiquitously expressed family of Rel-related transcription factors, is also involved in the regulation of autophagy (12, 13). It was reported that NF-κB activity promoted mammalian target of rapamycin (mTOR) activity to suppress autophagy (13). However, whether NF-κB is involved in regulation of mitophagy remains unknown.

MicroRNAs are functional small non-coding RNA molecules that regulate gene expression at a post-transcriptional level by targeting mRNAs for translational repression or degradation (14). miRNAs are crucial regulators of a variety of biological pathways involved in development, growth, homeostasis, immune response, and disease progression (15). Emerging evidence indicates that microRNAs are also involved in the regulation of autophagy-related pathways. For instance, miR-155–mediated autophagy accelerated the maturation of mycobacterial phagosome, thus decreasing the survival of intracellular mycobacteria in macrophages (16). The miR-302/367 cluster is located in intron 8 of *Larp7* gene, which is highly expressed in embryonic stem cells (17). Our previous study has identified that miR-302b is a crucial regulator of inflammatory response (18). Another study has revealed that mitophagy may be involved in host defense against *P. aeruginosa* invasion (19). Because a single miRNA may have multiple target genes, the specific role of miRNAs in regulation of mitophagy in bacterial infection remains an open question.

In the present study, we investigated the potential role of miR-302/367 cluster in modulating mitophagy and bacterial clearance in macrophages. Our study demonstrated that miR-302/367 cluster expression was significantly increased after *P. aeruginosa* infection. Overexpression of miR-302/367 cluster promoted mitophagy and enhanced the ability to scavenge ROS in macrophages, thus facilitating the elimination of intracellular *P. aeruginosa* and maintaining the cellular homeostasis. Importantly, we identified NF-κB as a novel functional target of miR-302/367 cluster in eliminating intracellular *P. aeruginosa*. These findings shed new light on host defense mechanisms in *P. aeruginosa* infection.

## Materials and Methods

### Ethics Statement

This research was performed in accordance with the Guide for the Care and Use of Laboratory Animals of the National Institutes of Health. The protocols of all animal studies were approved by the Institutional Animal Care and Use Committee (IACUC) at University of North Dakota (UND) (Assurance Number: A3917-01). The animal experimental procedures including treatment, care, euthanasia and endpoint choice followed the ARRIVE reporting guidelines. All experiments with human alveolar epithelial cells were approved by the UND Institutional Review Board Committee (IRB-200908-036, exempt 4) and have been conducted according to the principles expressed in the Declaration of Helsinki.

### Mice

C57BL/6J female and male mice (6–8 weeks) were obtained from the Jackson Laboratory (20). Animals were housed in a pathogen-free facility at the UND. The mice were divided randomly (3 mice per group) and were i.v. (intravenously) injected with control or miR-302a and miR-302b mimics (10 µg per mouse) for 24 h by in vivo-jetPEI reagent. The PA14 strain was grown for 16 h in LB broth at 37°C with shaking and was pelleted by centrifugation at 5000 g and resuspended in sterile PBS for infection. Twenty-four hours later, mice were anesthetized with 40 mg/kg and intranasally treated with 1 × 10^7^ CFU of PA14 in 50 µl PBS for 6 h. The BAL fluid, blood, and lungs from different groups mice were collected for CFU analysis of bacterial burdens. The infection experiments on mice model were performed three times independently.

### Cell Lines

Murine alveolar type II epithelial cells line MLE-12 (CRL-2110) and murine alveolar macrophage cell line MH-S (CRL-2019) were obtained from American Type Culture Collection and cultured following the manufacturer’s instructions (20). Human primary type II alveolar epithelial cells were isolated from a lung tissue of one healthy donor through assistance by Dr. Arvind Bansal and Dr. Tim Weiland at Altru Hospital and isolated as described previously (21) and only one donor was included in the experiments.

### Bacteria Preparation and Infection Experiments

The *P. aeruginosa* WT strain, PAO1, was kindly provided by Dr. S. Lory (Harvard University, Boston, MA) (22). The *P. aeruginosa* strain PA14 was kindly provided by Dr. George A O’Toole (Dartmouth Medical School) (23). PAK and PAO1-GFP were obtained from Dr. G. Pier (Channing Laboratory, Harvard Medical School, Boston, MA) (24). Bacteria were grown for 16 h in LB broth at 37°C with shaking. The bacteria were pelleted by centrifugation at 5000 g and resuspended in sterile PBS for infection. The infection experiments were performed according to a published protocol as previously described (18). Various mammalian cells were changed to antibiotic-free medium and infected by bacteria at an MOI of 10:1 bacteria-cell ratio.

### Transfection

siRNA specific for PHB2 was obtained from Invitrogen. NF-κB-expressing plasmid was a gift from Stephen Smale (Addgene plasmid #20018). miRNA-negative control (Ctrl-m), miR-302a mimics (302a-m), miR-302b mimics (302b-m), miRNA inhibitor negative control (NS-i), miR-302a inhibitor (302a-i), and miR-302b inhibitor (302b-i) were obtained from Dharmacon. MH-S cells were transfected with miRNA (50 nmol/L) or siRNA (50 nmol/L) using LipofectAmine 3000 reagent (Invitrogen) for 24 h following the manufacturer’s instruction. The ratio of transfection reagent to the plasmids was 1:1. Mice were i.v. administered with Ctrl-m or combined with 302a-m and 302b-m ((in vivo-jetPEI, Polyplus-transfection, 10 µl per mouse) for 24 h before bacteria challenge following the manufacturer’s instruction.

### Measurement of miRNA and mRNA Expression

Total RNA was extracted using the TRIzol (ThermoFisher) according to the manufacturer’s instructions. RNA was eluted in RNase-free water and stored at −80°C. Real-time PCR profiling of miRNAs was conducted on a SYBR Green-based, miScript PCR System (Qiagen). The expression of mRNAs was detected by QuantiTect SYBR Green RT-PCR Kit (Qiagen). The separate well 2-△△^Ct^ cycle threshold method was used to determine relative quantitative levels of individual miRNAs or mRNA and these were expressed as the fold difference to the sno202 or GAPDH (glyceraldehyde-3-phosphate dehydrogenase) (**Table S1**).

### Western Blotting Analysis

Mouse monoclonal antibodies (Abs) against GAPDH, PHB2, and LC3 were obtained from Santa Cruz Biotechnology; rabbit polyclonal Abs against LC3 was purchased from Abcam. The samples derived from cells and lung homogenates were lysed in RIPA buffer, separated by electrophoresis on 14% SDS–PAGE gels and transferred to nitrocellulose. Proteins were detected by Western blotting using primary Abs at a concentration of 1/500 and were incubated at 4°C overnight. Labeling of the first Abs was detected using relevant secondary Abs conjugated to HRP and detected using ECL reagents (Sigma-Aldrich).

### Confocal Microscopy

MH-S cells were transfected with miRNA-negative control or miR-302a and miR-302b mimics for 24 h. Cells were infected by PA14 at a MOI of 10:1 bacteria-cell ratio for 1 h, incubated with primary anti-LC3 Abs and anti-PHB2 Abs or anti cytochrome c Abs, and the second antibodies as described in the previous report (18, 25). 4′,6-diamidino-2-phenylindole (Sigma-Aldrich) was used to stain the nucleus.

### Luciferase Assay

MH-S cells were cultured in 24-well plates one day prior to transfection. pGL3 luciferase reporter plasmids (Promega) containing either a wild-type or mutated NF-κB 3′UTR were co-transfected with control or miR-302a and miR-302b mimic into MH-S cells with LipofectAmine 3000. Twenty-four hours after transfection, cell lysates were subjected to luciferase activity analysis by using the Luciferase Reporter Assay System (Promega) as previously described (18).

### Bacterial Internalization and Killing

Bacterial internalization and killing were measured as previously described (26). Briefly, the cells were infected with bacteria at a 10:1 MOI for 1 h. After 1 h of incubation at 37°C, the wells were washed and treated with 100 μg/ml polymyxin B (Sigma-Aldrich) for 1 h to kill extracellular bacteria. The cells were then washed in PBS and lysed in 0.2% Triton X-100 (Sigma Aldrich). The bacterial CFUs were determined by plating samples to LB agar plates.

### Phagocytosis Assay

The MH-S cells were plated in 96-well plates and grown overnight. The cells were treated with the serum-free medium for 1 h. Then GFP-PAO1 was used to infect the cells at 10:1 MOI. After an hour incubation at 37 °C, the wells were washed and treated with 100 μg/ml polymyxin B for 1 h to kill any remaining extracellular bacteria (18). The numbers of phagocytosed bacteria were counted using a Synergy HT fluorometer (BioTek) with 485 ± 20-nm excitation and 528 ± 20-nm emission filters. Background correction was done for autofluorescence. Triplicates were done for each sample and control.

### Oxidation Assays

To investigate the impact of miR-302/367 cluster on cell oxidation, the MH-S cells was collected after treatment as described above. Nitroblue tetrazolium (NBT) assay, dihydrodichlorofluorescein diacetate (H_2_DCF) assay and mitochondrial membrane potential (JC-1) assay were performed according to the manufacturer’s instructions, respectively (27). Triplicates were performed for each sample and control.

### Inflammatory Cytokine Profiling

Cytokine concentrations of IL-1β, IL-6, and TNF-α were measured by using ELISA kits (eBioscience). The medium supernatant was collected and 100 µl aliquots were added to the coated microtiter wells. The cytokine concentrations were determined with the corresponding detection HRP-conjugated Abs. The values were read at 450 nm as previously described (28).

### Transmission Electron Microscopy (TEM)

TEM was performed for identifying mitophagy induction by using modified process as previously described (29). Images were visualized using a Hitachi 7500 TEM and analyzed according to the previous published methods (29).

### Statistical Analysis

All statistical analyses were done with GraphPad Prism 5 software. Data are presented as mean ± SEM. Statistical analysis was performed by Student’s t-test for comparing two groups and by one-way analysis of variance (One Way ANOVA) with Tukey’s post hoc for multiple group comparisons. Differences in the mean values were considered to be significant at p < 0.05.

## Results

### miR-302/367 Cluster Facilitates the Killing of Intracellular *P. aeruginosa* In Vitro and In Vivo

**Figure 1:**
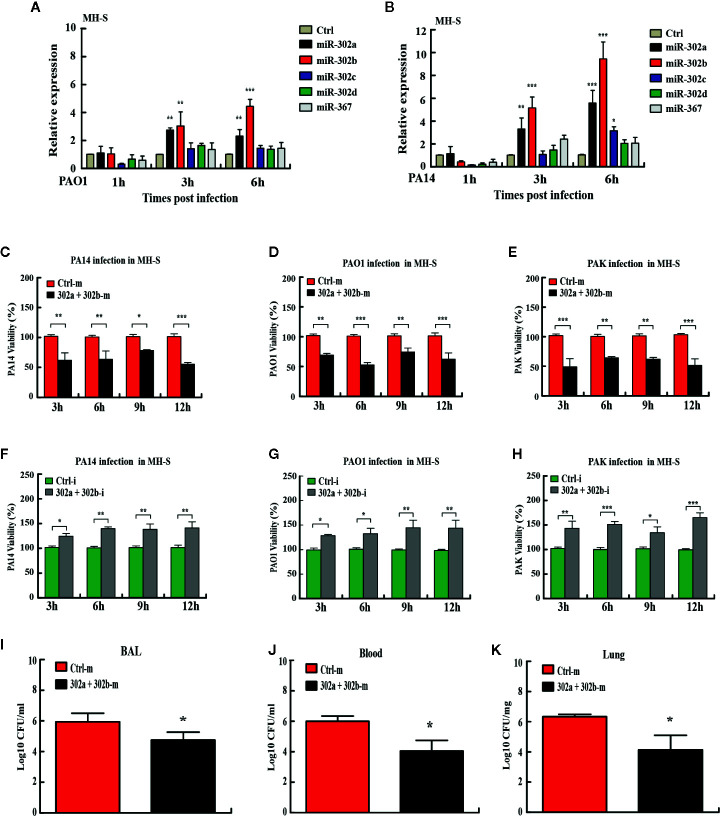
miR-302/367 cluster increases bacterial clearance in macrophages and in mice. **(A, B)** Time-dependent manner of miR-302/367 cluster expression in MH-S cells. MH-S cells were infected with PAO1 **(A)** or PA14 **(B)** at MOI 10:1 for 1 h and polymyxin B (100 µg/ml) was added for another 1 h to kill bacteria outside of the cells. The cell samples were also collected at different time points from 1 to 6 h. The miR-302/367 cluster expression in MH-S cells were detected by qRT-PCR. **(C**–**E)** MH-S cells were transfected with control (50 nmol/L) or miR-302a and 302b mimics (50 nmol/L) for 24 h followed by PA14 **(C)**, PAO1 **(D),** or PAK **(E)** infection at an MOI of 10:1 for 1 h, and intracellular bacterial clearance was determined by CFU assay at the indicated time points. **(F**–**H)** MH-S cells were transfected with control (50 nmol/L) or miR-302a and 302b inhibitors (50 nmol/L) for 24 h followed by PA14 **(F)**, PAO1 **(G),** or PAK **(H)** infection at an MOI of 10:1 for 1 h, and intracellular bacterial clearance was determined by CFU assay at the indicated time points. **(I**–**K)** The mice were i.v. (intravenously) injected with control or miR-302a and miR-302b mimics (10 µg per mouse). Twenty-four hours later, mice were treated with 1 × 10^7^ CFU of PA14 for 6 h. BAL fluid **(I)**, blood **(J)**, and lungs **(K)** were collected for CFU analysis of bacterial burdens. Data are shown as the mean ± SEM of three independent experiments. *p < 0.05; **p < 0.01, ***p < 0.001.

miRNAs have been reported as vital regulators of gene expression to influence diverse biological processes at the posttranscriptional level (30). Previously, the studies have not considered the miRNA as a cluster for coordinated and collective activities in bacterial respiratory infections. To determine whether the expression of the miR-302/367 cluster is altered, we performed quantitative PCR (qPCR) on RNA lysates from mouse alveolar macrophage MH-S cells or murine lung epithelial MLE-12 cells after *P. aeruginosa* infection. Expression of miR-302d and miR-367 was not statistically different between *P. aeruginosa*-infected and control cells, whereas miR-302a and miR-302b expression was significantly elevated with *P. aeruginosa* infection at 3 h and 6 h **(** **Figures 1A, B** **)**. Interestingly, members of the cluster exhibited a similar trend towards up-regulation in MLE-12 cells **(** **Figures S1A, B** **)**. These findings suggest that both miR-302a and miR-302b may be involved in the process of host defense during *P. aeruginosa* infection and are chosen as main targets for next studies.

To define the collective role of the miR-302/367 cluster in *P. aeruginosa* infection, we next examined its effects on the clearance of *P. aeruginosa* by colony-forming unit (CFU) assay. MH-S cells were transiently transfected with chemically-synthesized miR-302a and 302b mimics or inhibitors and then challenged with strain PA14. Our results showed that enforced expression of miR-302a and 302b significantly increased clearance of intracellular PA14 in MH-S cells from 3 h to 12 h post infection **(** **Figure 1C** **)**. To validate these data, we tested additional *P. aeruginosa* strains. The clearance of intracellular PAO1 and PAK **(** **Figures 1D, E** **)** in MH-S cells transfected with miR-302a and 302b mimics were also increased at the indicated time points post infection. However, transfection with miR-302a and 302b inhibitors decreased bacterial clearance of PA14 in MH-S cells **(** **Figure 1F** **)**. Similarly, the clearance of PAO1 and PAK **(** **Figures 1G, H** **)** also were declined in miR-302a and 302b inhibitors-treated MH-S cells. Interestingly, transfection with miR-302/367 cluster mimics also showed antibacterial effects with decreased CFU in mice model following PA14 infection **(** **Figures 1I–K** **)**. Thus, these data indicate that miR-302/367 cluster facilitates the killing of intracellular *P. aeruginosa* in vitro and in vivo.

### miR-302/367 Cluster Regulates ROS Production in Macrophages

**Figure 2:**
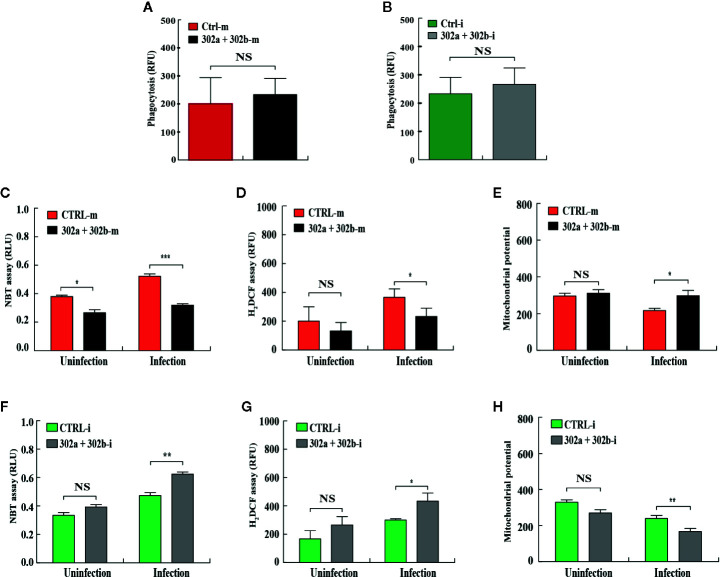
miR-302/367 cluster regulates ROS production in macrophages. **(A, B)** MH-S cells were transfected with control or miR-302a and 302b mimics **(A)**, control or miR-302a and 302b inhibitors **(B)**. Twenty-four hours later, cells were infected with PAO1-GFP at MOI 10:1 for 1 h. Fluorescence intensity was calculated from triplicate samples **(A, B)**. **(C**–**H)** MH-S cells were transiently transfected with control or miR-302a and miR-302b mimics **(C**–**E)**, control or miR-302a and 302b inhibitors **(F**–**H)** for 24 h, and then either left uninfected or infected with PA14 for 1 h. Superoxide production in MH-S cells was detected by an NBT assay at a wavelength of 560 nm **(C, F)** and an H_2_DCF assay at a wavelength of 488 nm **(D, G)**. Mitochondrial potential of MH-S cells was measured by JC-1 fluorescence assay at a wavelength of 532 nm **(E, H)**. Data are shown as the mean ± SEM of three independent experiments. *p < 0.05; **p < 0.01; ***p < 0.001. NS, not significant.

To test whether miR-302/367 cluster affects phagocytosis in macrophages after *P. aeruginosa* infection, the MH-S cells were transfected with miR-302a and 302b mimics or inhibitors, respectively. Our results showed that neither overexpression nor inhibition of miR-302/367 cluster had an effect on phagocytosis ability after infection compared with control cells **(** **Figures 2A, B** **)**. In addition to phagocytosis, reactive oxygen species (ROS) and inflammatory cytokines are also involved in intracellular killing during bacterial infection (31). To measure whether miR-302/367 cluster affects oxidative stress in macrophages after *P. aeruginosa* infection, MH-S cells were transfected with miR-302a and 302b mimics or inhibitors for 24 h, and then either left uninfected or infected with PA14 for 1 h. As determined by nitroblue tetrazolium (NBT) assay **(** **Figure 2C** **)**, MH-S cells showed approximately 2-fold decrease in oxidative stress after transfected with miR-302a and miR-302b mimics compared to control mimics-transfected cells. This result was confirmed by dihydrodichlorofluorescein diacetate (H_2_DCF) assay that quantifies superoxide **(** **Figure 2D** **)**. Furthermore, we found that a decreased mitochondrial membrane potential was shown in PA14-infected MH-S cells while transfected with miR-302a and miR-302b mimics restored these changes, as determined using a JC-1 fluorescence assay (32) **(** **Figure 2E** **)**. However, oxidative stress was increased **(** **Figures 2F, G** **)** and mitochondrial membrane potential **(** **Figure 2H** **)** was declined after transfected with miR-302a and miR-302b inhibitors compared to control inhibitors-transfected cells. Altogether, these findings suggest that miR-302/367 cluster could regulate ROS production during *P. aeruginosa* infection in macrophages.

### miR-302/367 Cluster Induces Mitophagy in Macrophages

**Figure 3:**
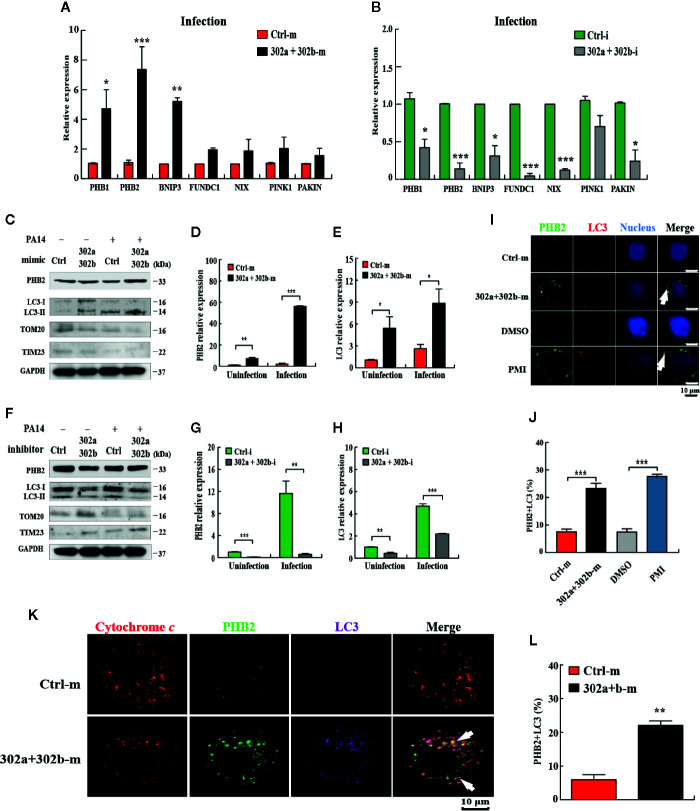
miR-302/367 cluster induces mitophagy in macrophages. **(A–H)** MH-S cells were transfected with miR-302a and miR-302b mimics or inhibitors for 24 h and then either left uninfected or infected with PA14 for 6 h. Expression level of mitophagy receptors or regulators in MH-S cells were detected by qPCR **(A, B)**. The mitophagy activity was detected by Western blotting **(C, F)** and expression levels of PHB2 **(D, G)** and LC3 **(E, H)** were detected by qPCR. **(I)** MH-S cells were transfected with control or miR-302a and 302b mimics for 24 h and reacted with mouse anti-PHB2 antibody followed by Alexa Fluor 488-conjugated goat anti-mouse IgG antibody (green). Endogenous LC3 was stained with LC3 antibody followed by Alexa Fluor 594-conjugated goat anti-rabbit IgG antibody (red). Cells treated with p62-mediated mitophagy inducer (PMI) were used as a positive control. **(K)** MH-S cells were fixed and then immunostained by mitochondrial protein cytochrome *c* (red) with counterstaining of PHB2 (green) or LC3 (purple). The co-localization of PHB2 with LC3 was detected by confocal microscopy. Arrows indicate the co-localization of PHB2 with LC3; scale bar, 10 μm. **(J, L)** Quantification of the co-localization of PHB2 with LC3-positive autophagosomes is shown. Data are shown as the mean ± SEM of three independent experiments. *p < 0.05; **p < 0.01; ***p < 0.001.

Given the current evidence regarding mitophagy regulation in *P. aeruginosa* infection (19) and miR-302/367 cluster down-regulated ROS production, we hypothesized that miR-302/367 cluster may play a role in modulating the mitophagy process during *P. aeruginosa* infection. To test the hypothesis that miR-302/367 cluster induces mitophagy in macrophages, we first detected the expression level of the main mitophagy receptors PHB1 (prohibitin 1), PHB2 (prohibitin 2), BNNIP3 (BCL2 and adenovirus E1B 19-kDa-interacting protein 3), FUNDC1 (*FUN14* domain containing 1) and NIX (BNIP3-like) or regulators PINK1 (PTEN induced putative kinase 1) and PAKIN in MH-S cells by qPCR. We found that miR-302/367 cluster significantly regulated expression of PHB2 compared to other mitophagy receptors or regulators in MH-S cells **(** **Figures 3A, B** **)**. Thus, we focused on PHB2 as a main mitophagy marker in this study.

Next, we evaluated the mitophagy activity in MH-S cells by testing the expression of PHB2, TOM20 (Translocase outer membrane 20), TIM23 (Translocase inner membrane 23) (33) and processing of LC3. qPCR data showed that transfection with miR-302a and miR-302b mimics significantly increased the levels of miR-302a and miR-302b **(** **Figures S1C, D** **)**, whereas transfection of miR-302a and miR-302b inhibitor markedly decreased miR-302a and miR-302b levels in MH-S cells **(** **Figures S1E, F** **)**. Western blotting results showed that transfection of miR-302a and miR-302b mimics partially increased the amount of LC3-II and PHB2 and decreased the amount of TOM20 or TIM23 in control and PA14-infected MH-S cells **(** **Figure 3C** **)**. However, transfection of miR-302a and miR-302b inhibitors blocked mitophagy activity with reduced PHB2 and LC3-II (or impeded the degradation of TOM20 and TIM23) in MH-S cells before and after PA14 challenge **(** **Figure 3F** **)**. Likewise, PHB2 and LC3 mRNA levels were increased in MH-S cells transfected with miR-302a and miR-302b mimics **(** **Figures 3D, E** **)**, and decreased after transfection with miR-302a and miR-302b inhibitors **(** **Figures 3G, H** **)**. In addition, we also showed that PA14 infection enhanced mitophagy *in vitro* and *in vivo* **(** **Figures S2A–C** **)** and confirmed the mitophagic receptor expression induced by each single miRNA of miR-302/367 cluster **(** **Figures S2D–F** **)**. Surprisingly, enforced expression of miR-302/367 cluster also increased miR-302a and miR-302b expression **(** **Figures S3A–D** **)** and induced the expression of mitophagy receptor in human primary alveolar epithelial cells **(** **Figures S3E–3H** **)**, which suggesting that the effects within the miR-302/367 cluster on mitophagy may be relevant to human infectious disease. Furthermore, the results from confocal microscopy showed that miR-302a and miR-302b overexpression elevated the co-localization of PHB2 with endogenous LC3 autophagosomes **(** **Figures 3I, J** **)**. Immunostaining with specific antibodies against cytochrome *c* **(** **Figures 3K, L** **)** further confirmed induction of mitophagy by miR-302a and miR-302b. Importantly, immune transmission electronic microscope (TEM) showed the anti-LC3 gold-labeled dots around mitochondria regions after transfection of miR-302a and miR-302b mimics **(** **Figure S2G** **)**. Taken together, these data demonstrate that miR-302/367 cluster augments mitophagy response in macrophages.

### miR-302/367 Cluster Induces Mitophagy and Alters Oxidation to Eliminate Intracellular *P. aeruginosa*

**Figure 4:**
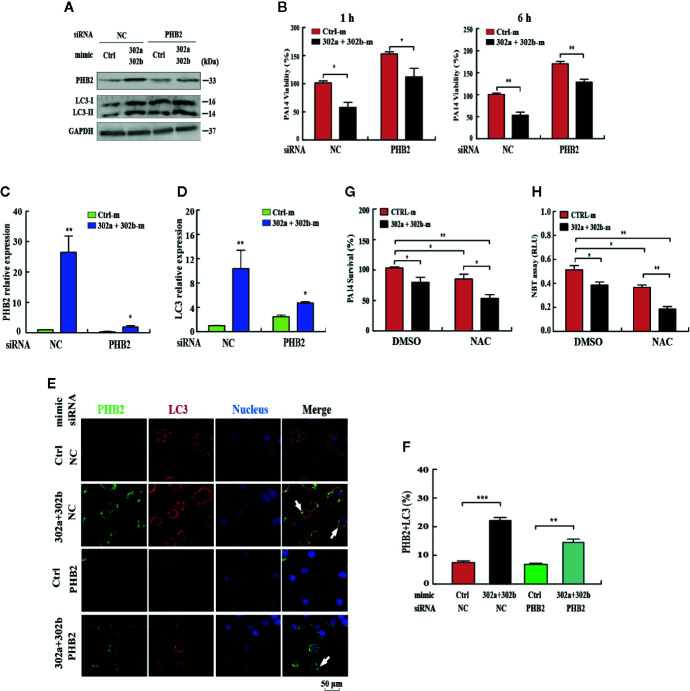
miR-302/367 cluster-induced mitophagy promotes the elimination of invading *P. aeruginosa*. **(A–F)** MH-S cells were transiently co-transfected with control or miR-302a and miR-302b mimics together with a control siRNA or PHB2 siRNA. The expression levels of PHB2 and LC3 were detected by Western blotting **(A)**. Intracellular *P. aeruginosa* clearance was determined by CFU assay at the indicated time after challenging with PA14 for 1 h **(B)**. The mRNA levels of PHB2 **(C)** and LC3 **(D)** were detected by qPCR. The co-localization of PHB2 with LC3 was detected by confocal microscopy **(E)**. Arrows indicate the co-localization of PHB2 with LC3; scale bar, 50 μm. Quantification of the co-localization of PHB2 with LC3-positive autophagosomes is shown **(F)**. **(G, H)** MH-S cells were transfected with control or miR-302a and miR-302b mimics for 24 h. Then the cells were pretreated with NAC (3mM) for 2 h and infected with PA14 at an MOI 10:1 for 1 h. Invading bacterial clearance was determined by CFU assay **(G)**. Superoxide production in MH-S cells was detected by an NBT assay as previously shown **(H)**. Data are shown as the mean ± SEM of three independent experiments. *p < 0.05; **p < 0.01; ***p < 0.001.

To determine whether the miR-302/367 cluster impacts the elimination of internalized *P. aeruginosa* via mitophagy, we blocked mitophagy by specific siRNAs against PHB2. Transfection with miR-302a and miR-302b mimics enhanced mitophagy and reduced the viability of *P. aeruginosa* in MH-S cells, and these effects were partly reversed by inhibition of PHB2, as indicated by Western blotting **(** **Figure 4A**, **Figure S2H** **)** and CFU assays **(** **Figure 4B** **)**. In addition, PHB2 and LC3 mRNA levels were dramatically decreased in MH-S cells transfected with PHB2 siRNA vs. control siRNA **(** **Figures 4C, D** **)**. The confocal microscopy results showed that overexpression of miR-302a and miR-302b enhanced the co-localization of PHB2 with LC3-positive autophagosomes in MH-S cells transfected with PHB2 siRNA vs. control siRNA **(** **Figures 4E, F** **)**. In addition, transfection with miR-302a and miR-302b mimics significantly reduced the survival of *P. aeruginosa* in MH-S cells as indicated by CFU data and decreased oxidative stress by NBT assay. These effects were partly enhanced by treatment with *N*-acetyl-_L_-cysteine (NAC), a widely-used inhibitor of ROS **(** **Figures 4G, H** **)**. Thus, these findings demonstrate that miR-302/367 cluster promotes the elimination of *P. aeruginosa* by activating mitophagy in macrophages.

### miR-302/367 Cluster Post-Transcriptionally Suppresses NF-κB by Interacting With Its 3′ UTR

**Figure 5:**
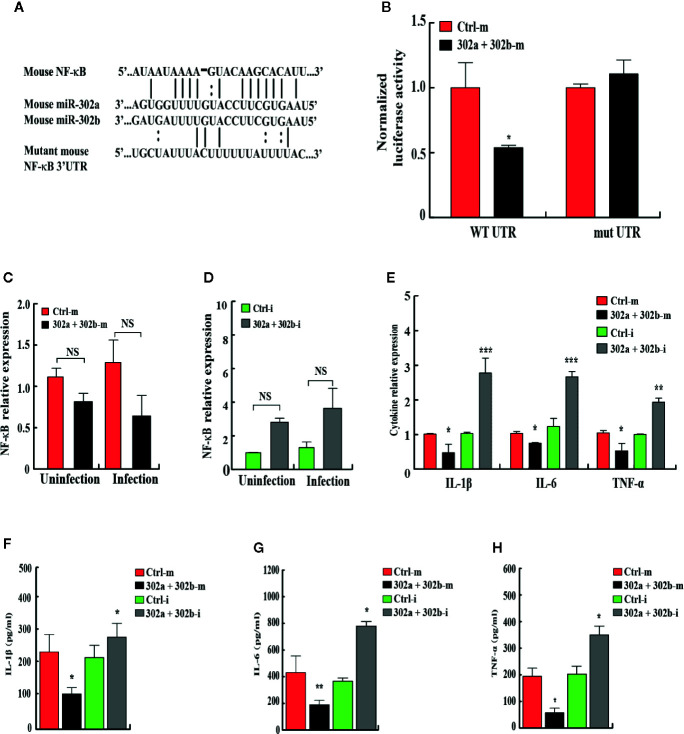
miR-302/367 cluster post-transcriptionally represses NF-κB expression by targeting its 3′UTR. **(A)** Sequences of mouse miR-302a and their predicted interactions with conserved miR-302a seeds found within the 3′UTRs of NF-κB are shown. The sequence of the NF-κB 3′UTR seed mutant used for the reporter assays and the predicted disruption of the miR-302a interaction are also shown. **(B)** Normalized luciferase activity of a reporter containing the wild-type or point-mutated 3′UTR reporter constructs (wt. UTR or mutant UTR) of NF-κB in MH-S cells co-transfected with control mimics or miR-302a and miR-302b mimics. **(C, D)** MH-S cells were transfected with miR-302a and miR-302b mimics **(C)** or inhibitors **(D)** for 24 h and then either left uninfected or infected with PA14 for 6 h. The expression levels of NF-κB mRNA were detected by qPCR **(C, D)**. The expression levels of different cytokine mRNA were detected by qPCR **(E)**. The medium supernatant was collected and different cytokines were measured by a standard ELISA **(F–H)**. Data are shown as the mean ± SEM of three independent experiments. *p < 0.05; **p < 0.01; ***p < 0.001; NS, not significant.

To identify the novel target of miR-302/367 cluster that modulates mitophagy, bioinformatics analysis was performed with TargetScan (http://www.targetscan.org/) and mirnatargets (http://www.mirdb.org/). We found that NFkB1 (p50) subunit of NFkB family displayed a potential seed match for miR-302a and miR-302b in its 3′-untranslated region (3′UTR) and constructed a luciferase reporter plasmids containing either the wild type or mutant seed regions **(** **Figure 5A** **)**. MH-S cells were co-transfected with scrambled control mimics or miR-302a and miR-302b mimics together with these NF-κB luciferase reporter constructs. Overexpression of miR-302a and miR-302b mimics inhibited the expression of luciferase activity reflecting expression of the construct that was fused to the WT NF-κB 3′UTR (p < 0.05), but failed to repress the activity of the luciferase construct containing the 3′UTR site mutations of NF-κB **(** **Figure 5B**, **Table S1** **)**. To explore whether miR-302/367 cluster represses endogenous NF-κB, MH-S cells were transfected with control mimics or miR-302a and miR-302b mimic. The results showed that in both gain- and loss-of-function studies, miR-302a and miR-302b had no specific effect on the levels of NF-κB mRNA expression **(** **Figures 5C, D** **)**. We further measured cytokine concentrations in MH-S cells 24 h post-PA14 infection to gauge the inflammatory response. IL-1β, IL-6, and TNF-α levels were significantly decreased in miR302a and 302b mimics-transfected MH-S cells compared to control mimic-transfected cells **(** **Figures 5E–H** **)**. In contrast, in cells transfected with miR-302a and miR-302b inhibitors, the protein and mRNA levels of IL-1β, IL-6, and TNF-α were increased **(** **Figures 5E–H** **)**, suggesting that NF-κB was activated in control MH-S cells after PA14 infection but not in the combination of miR-302a and miR-302b mimics-transfected MH-S cells. Altogether, these results indicate that miR-302/367 cluster post-transcriptionally represses the expression of NF-κB by interacting with its 3′UTR seed region.

### miR-302/367 Cluster Promotes Mitophagy to Maintain the Cellular Homeostasis by Regulating NF-κB During *P. aeruginosa* Infection

To determine whether the miR-302/367 cluster impacts the elimination of internalized *P. aeruginosa.*

**Figure 6:**
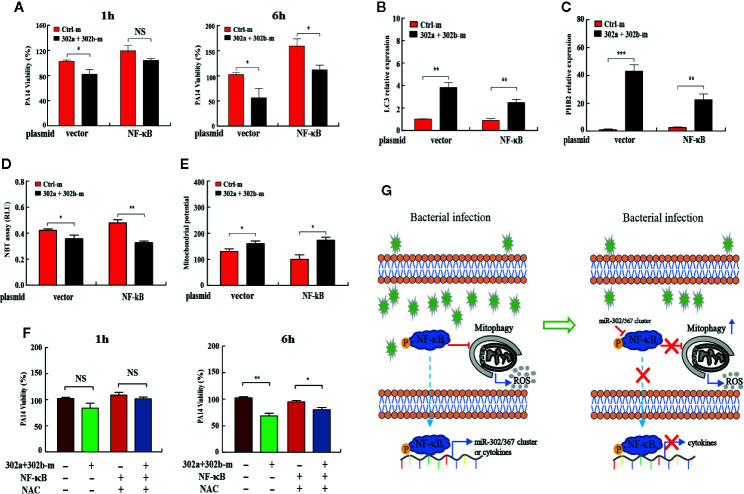
miR-302/367 cluster promotes mitophagy against *P. aeruginosa* through regulation of NF-κB.** (A**–**E)** MH-S cells were co-transfected with control or miR-302a and miR-302b mimic together with a control plasmid or a plasmid expressing NF-κB for 24 h, and then infected with PA14 for 1 h. Intracellular bacterial viability was determined by CFU assay at the indicated time points **(A)**. The expression levels of LC3, and PHB2 mRNA were detected by qPCR **(B, C)**. Superoxide production in MH-S cells was detected by an NBT assay **(D)** and mitochondrial potential of MH-S cells was measured by JC-1 fluorescence assay **(E)** as previously shown. **(F)** MH-S cells were co-transfected with controls or miR-302a and miR-302b mimic together with a control plasmid or a plasmid expressing NF-κB for 24 h, and then pretreated with NAC (3 mM) for 2 h and infected with PA14 at an MOI 10:1 for 1 h. Invading bacterial clearance was determined by CFU assay at the indicated time points. **(G)** Schematic model of the critical role of miR-302/367 cluster in respiratory bacterial infection pathogenesis. miR-302/367 cluster functions as a negative feedback regulator in mitophagy induction by targeting NF-κB. Data are shown as the mean ± SEM of three independent experiments. *p < 0.05; **p < 0.01; ***p < 0.001; NS, not significant.

Next, to investigate whether miR-302/367 cluster promotes mitophagy-induced bacterial elimination by targeting NF-κB, we tested the role of NF-κB on mitophagy in MH-S cells. MH-S cells were co-transfected with miR-302a and miR-302b mimics and NF-κB-expressing plasmid, and then detected the intracellular bacterial load. Interestingly, CFU assay indicated that overexpression of miR-302/367 cluster significantly decreased the bacterial load at 6 h post-infection, whereas NF-κB overexpression significantly inhibited miR-302/367 cluster-mediated bacterial clearance in MH-S cells **(** **Figure 6A** **)**. In addition, transient transfection with a plasmid expressing NF-κB effectively downregulated the expression of LC3 and PHB2 when compared to treatment with the control plasmid **(** **Figures 6B, C** **)**. These data indicate that NF-κB overexpression dramatically inhibits mitophagy in macrophages. As determined by NBT assay **(** **Figure 6D** **)** and JC-1 fluorescence assay **(** **Figure 6E** **)**, oxidative stress and mitochondrial membrane potential were both impacted by the addition of NF-κB plasmid compared to control cells, which could also off-set the effects by miR-302a and miR-302b mimics. Furthermore, MH-S cells were co-transfected with miR-302a and miR-302b mimics and NF-κB-expressing plasmid, and then treated with NAC to evaluate intracellular bacterial loads. CFU assay indicated that expression of NF-κB dampened the bacterial clearance, while overexpression of miR-302/367 cluster or adding NAC decreased intracellular bacterial loads by scavenged excessive ROS compared to the relevant control treatments **(** **Figure 6F** **)**. Collectively, these results suggest that miR-302/367 cluster promotes mitophagy-mediated elimination of *P. aeruginosa* by regulating NF-κB and reducing ROS accumulation to maintain the cellular homeostasis **(** **Figure 6G** **)**.

## Discussion

Mitophagy has been demonstrated to play an essential role in the host immune response against *P. aeruginosa* infection (19). However, the underlying molecular mechanism involved in mitophagy-mediated clearance of *P. aeruginosa* remains largely unknown. Here we report a novel role of the miR-302/367 cluster in regulating mitophagy and *P. aeruginosa* elimination in macrophages by targeting NF-κB, which sheds light on understanding of the host defense against *P. aeruginosa* infection.

Accumulating evidence has shown that the miR-302/367 cluster is highly expressed in embryonic stem cells and plays a crucial role in their self-renewal and somatic cell reprogramming (34, 35). The targets of these miRNAs regulate various cellular processes (36–38). However, our study is the first to show that miR-302/367 cluster participates in cell crosstalk between respiratory bacterial infection and mitophagy. This study shows that miR-302/367 cluster expression is enhanced after *P. aeruginosa* infection, which is consistent with the previous study showing that miR-302b, a member of the miR-302/367 cluster, responded to *P. aeruginosa* and *Klebsiella pneumoniae* challenge to regulate inflammatory response (18). These data implicate a critical link of miR-302/367 cluster with gram-negative bacterial infection.

As one of the most essential cell types in the antibacterial immunity, macrophages function as the predominant cell for making response to *P. aeruginosa* infection (18, 27). The cells uptake and subsequently kill invasive bacteria by regulating key factors of pattern recognition receptors (PRR) in recognition of PAMP, but also initiating an inflammatory response against *P. aeruginosa* infection (39, 40). Therefore, precise modulation of macrophage activity is crucial for immune defense against *P. aeruginosa* infection. The previous study has demonstrated that miR-302b regulates the production of proinflammatory cytokines in macrophages in response to *P. aeruginosa* invasion (18), indicating that miR302s might be required for the macrophage-mediated immune defense against infection. In the present study, MH-S cells were chosen as the relevance to pulmonary innate immune response to *P. aeruginosa*. The results demonstrate that miR-302/367 cluster decreased the survival of different intracellular *P. aeruginosa* strains at all tested time points, indicating that miR-302/367 cluster promoted macrophage-mediated bacterial elimination. The observation is similar to other miRNAs showing that miR-155 promoted the maturation of mycobacterial phagosome and decreased the survival of intracellular BCG in RAW264.7 cells (16). Although different *P. aeruginosa* strains might cause different host response regards to their virulence and interaction with the host cells, the data obtained from the current study could be attributable to the macrophage-triggered immune response to reduce intracellular bacterial survival.

Mitophagy and the mitochondria-mediated pathways have been implicated in a crucial process of the innate immune response against various pathogens, including *P. aeruginosa* (6, 19, 41, 42). However, the mechanism by which mitophagy impacts the bacterial infection is largely unclear. A recent study has identified an inner mitochondrial membrane protein, PHB2, as a key mitophagy receptor for paternal mitochondrial clearance in *Caenorhabditis elegans* (29). In the present study, we demonstrate that miR-302/367 cluster induces the processing of LC3 and the accumulation of LC3 and PHB2 puncta in both *P. aeruginosa*-challenged and unchallenged MH-S cells, indicating that miR-302/367 cluster accelerates mitophagic response in macrophages and reduces intracellular bacterial loads. In addition, we found that inhibition of mitophagy with PHB2 siRNA reduced miR-302/367 cluster-mediated mitophagy and bacterial elimination. These findings indicate that mitophagy induced by infection is regulated by a unique set of microRNAs, the miR-302/367 family.

In view of recent studies showing that intracellular infection with several pathogens such as *Listeria monocytogenes* results in altered host mitochondria dynamics (42), it is assumed that dysfunction of mitochondria may serve as a signal for intracellular infection and targeting mitophagy may be a potential therapeutic strategy to treat *P. aeruginosa*. Interestingly, our results showed that inhibition of mitophagy by PHB2 siRNA attenuated, but not fully blocked the miR302s-mediated effect on *P. aeruginosa* invasion, indicating that other alternative mechanisms may be involved in the miR302s-mediated bacterial elimination. Studies have demonstrated that macrophages also employ an oxygen-dependent system to control intracellular pathogens (43, 44). We found that mitophagy activation may positively contribute to defense against *P. aeruginosa* infection by regulating ROS production. Our fresh data showed that overexpression of miR-302/367 cluster enhanced mitophagy activation and decreased the production of ROS in *P. aeruginosa*-infected MH-S cells, suggesting that miR-302/367 cluster may exert antibacterial function through protected host cell from ROS-mediated damage and maintained the intracellular homeostasis such as mitochondrial homeostasis. However, the exact mechanism by which miR-302/367 cluster regulates the effect of ROS-mediated damage on host cell remains to be explored.

Strikingly, our bioinformatic studies found that NF-κB mRNA has a 7mer-A1 seed match in its 3′UTR, which predicts NF-κB as a novel target for the miR-302/367 cluster. In unstimulated cells, NF-κB is sequestered in the cytoplasm by binding to inhibitory κB proteins (IκB) (45). In response to a variety of stimuli, such as oncogenes and inflammatory cytokines, the proteasome-dependent degradation of IκB allows the translocation of NF-κB to the nucleus and its binding to the promoter region of target genes involved in the control of different cellular responses, including apoptosis and autophagy/mitophagy (11, 13, 40, 46). Our study indicates that miR-302/367 cluster post-transcriptionally down-regulates NF-κB expression, thus augmenting mitophagy in macrophages in an infection event. In addition, CFU assay suggests that overexpression of NF-κB reduces intracellular bacterial clearance when transfected with miR-302s mimics, indicating that NF-κB is the key target of the miR-302/367 cluster in modulating the mitophagy response and bacterial elimination in macrophages. These findings characterize a new crosstalk between host microRNA response and mitophagic activity required for defending pathogens’ invasion, suggesting that *P. aeruginosa*-induced miR-302/367 cluster targets NF-κB, a key regulator of mTOR signaling and is crucial in sensing and responding to cellular stimuli. Not limiting in macrophages, our assessment in primary human epithelial cells and mouse epithelial cell lines indicates the same role of the miR-302/367 cluster in host defense against infection, warranting a further investigation for epithelial cells’ involvement. Given that mitophagy also plays a key role in host metabolism, miR-302/367 cluster may have broad roles in modulating the metabolic pathways in multiple types of host cells.

In summary, the current studies demonstrate that the miR-302/367 cluster is induced by *P. aeruginosa* infection, and promotes mitophagy in macrophages, conferring protection against infection of *P. aeruginosa*. Importantly, we identify the chief mechanism of this bacterial clearance involving ROS reduction rather than phagocytosis and the oxidative stress regulation is dependent on mitophagy-mediated pathways. Further, we illustrated that the miR-302/367 cluster targets at a critical negative mitophagy regulator and inflammatory master controller NF-κB to influence *P. aeruginosa* viability during its infection in macrophages. Although some details by which *P. aeruginosa* gets access to mitochondria and impacts mitophagy induction needs further characterization, it is likely to consider both miRNAs and host mitophagy as potential targets for developing therapeutic interventions against *P. aeruginosa*-related diseases.

## Data Availability Statement

All datasets presented in this study are included in the article/**Supplementary Material**.

## Ethics Statement

The studies involving human participants were reviewed and approved by the UND Institutional Review Board Committee (IRB-200908-036, exempt 4). Written informed consent for participation was not required for this study in accordance with the national legislation and the institutional requirements. The animal study was reviewed and approved by the Institutional Animal Care and Use Committee (IACUC) at University of North Dakota (UND) (Assurance Number: A3917-01).

## Author Contributions

TH, BY, and MW designed the experiments. TH, QP, CZ, and PG performed the experiments. TH and QP analyzed the data. PL, XZ, and YC contributed new reagents or analytic tools. TH, QP, and MW wrote the paper. All authors contributed to the article and approved the submitted version.

## Funding

This project was supported by National Natural Science Foundation of China (No. 31900120) and China Scholarship Council (201606240043) to TH. This project was also supported by National Key Program of Research and Development, Ministry of Science and Technology (China) (2016YFC0503200) to BY, NIH grant R01 AI109317 and AI138203-01 to MW, NIH grant 5P20GM113123 to the UND imaging core, respectively.

## Conflict of Interest

The authors declare that the research was conducted in the absence of any commercial or financial relationships that could be construed as a potential conflict of interest.

## Supplementary Material

Supplementary Figure 1
*P. aeruginosa* infection robustly increases expression of miR-302/367 cluster in MLE-12 cells or MH-S cells. **(A and B)** Time-dependent manner of miR-302/367 cluster expression in MLE-12 cells. MLE-12 cells were infected with PAO1 **(A)** or PA14 **(B)** at MOI 10:1 for 1 h and polymyxin B was added for another 1 h to kill bacteria outside of the cells. The cell samples were also collected at different time points from 1 to 6 h. The miR-302/367 cluster expression in MLE-12 cells were detected by qRT-PCR. **(C-F)** MH-S cells were transfected with miR-302a and miR-302b mimics or inhibitors for 24 h and then either left uninfected or infected with PA14 for 6 h. Expression levels of miR-302a **(C and E)** and miR-302b **(D and F)** were detected by qPCR. The data are representative of three experiments and are shown as means ± SEM. *, p < 0.05; **, p < 0.01; ***, p < 0.001.

Supplementary Figure 2
*P. aeruginosa* infection and miR-302/367 cluster induces expression of mitophagy receptor in *in vitro* and *in vivo*. **(A**–**C)** MH-S cells were infected with PA14 at MOI 10:1 for 1 h and polymyxin B was added for another 1 h to kill bacteria outside of the cells **(A and C)**. The cell samples were also collected at 6 h. Mice were treated with 1×10^7^ CFU of PA14 and the lung tissues samples (n = 3) were collected at 6 h **(B)**. The expression level of PHB2 and LC3 in MH-S cells were detected by qPCR (A) and Western blotting **(C)**. The expression level of PHB2 and LC3 in lung tissues samples were detected by qPCR **(B)**. **(D-F)** The MH-S cells were transfected with each single miRNA of miR-302/367 cluster for 24 h. Expression levels of LC3 and PHB2 were detected by Western blotting **(D)** and qPCR **(E and F**). **(G)** Electron micrographs of MH-S cells stained with immunogold-conjugated anti-LC3 antibody to detect LC3. Cells were transfected with control or miR-302a and miR-302b mimics. Black arrows, immunogold particles labeling LC3. Mito, mitochondrion; AL, autolysosome. Scale bars, 1 μm. (**H**) Densitometric quantification of the Western blotting gel data from **Figure 4A** was used by Quantity one software and is shown as means. Data are shown as the mean ± SEM of three independent experiments. *P < 0.05; **, p < 0.01; ***, p < 0.001.

Supplementary Figure 3 miR-302/367 cluster induces expression of mitophagy receptor in human primary alveolar epithelial cells. **(A**–**H)** The human primary alveolar epithelial cells were transfected with miR-302a and miR-302b mimics **(A, B, E, and F)** or inhibitors **(C, D, G, H)** for 24 h and then either left uninfected or infected with PA14 for 6 h. Expression levels of miR-302a and miR-302b **(A**–**D)** or LC3 and PHB2 **(E**–**H)** were detected by qPCR. Data are shown as the mean ± SEM of three independent experiments. *P < 0.05; **, p < 0.01; ***, p < 0.001.
