# Supplementary figures and images for: MicroRNA-302/367 Cluster Impacts Host Antimicrobial Defense via Regulation of Mitophagic Response Against Pseudomonas aeruginosa Infection
Source: Front Immunol. 2020 Oct 7;11:569173. doi: 10.3389/fimmu.2020.569173 (PMC7576609; doi:10.3389/fimmu.2020.569173)

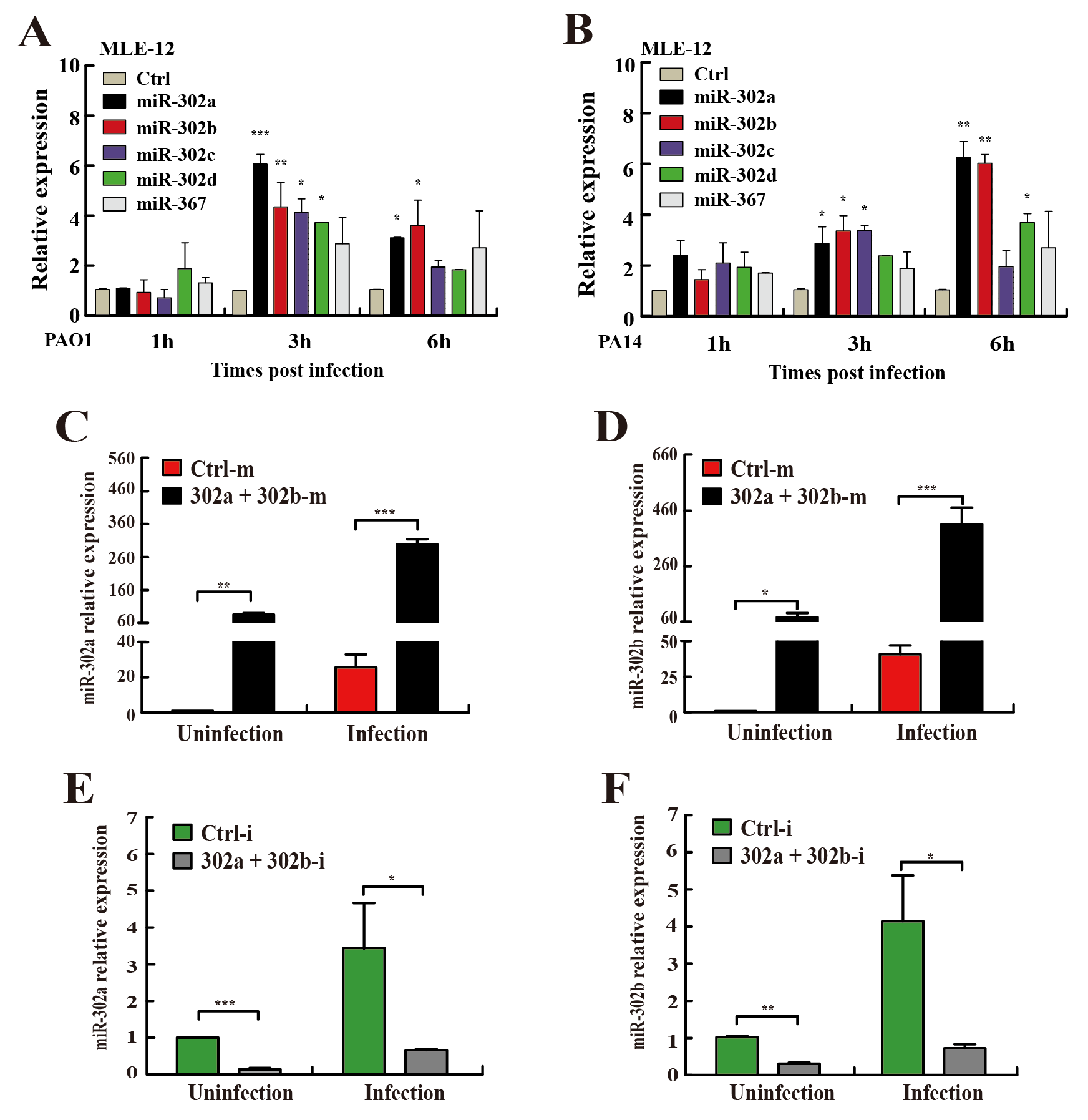

Supplement: Supplementary Figure 1 — P. aeruginosa infection robustly increases expression of miR-302/367 cluster in MLE-12 cells or MH-S cells. (A and B) Time-dependent manner of miR-302/367 cluster expression in MLE-12 cells. MLE-12 cells were infected with PAO1 (A) or PA14 (B) at MOI 10:1 for 1 h and polymyxin B was added for another 1 h to kill bacteria outside of the cells. The cell samples were also collected at different time points from 1 to 6 h. The miR-302/367 cluster expression in MLE-12 cells were detected by qRT-PCR. (C-F) MH-S cells were transfected with miR-302a and miR-302b mimics or inhibitors for 24 h and then either left uninfected or infected with PA14 for 6 h. Expression levels of miR-302a (C and E) and miR-302b (D and F) were detected by qPCR. The data are representative of three experiments and are shown as means ± SEM. *, p < 0.05; **, p < 0.01; ***, p < 0.001. [file Image_1.tif]

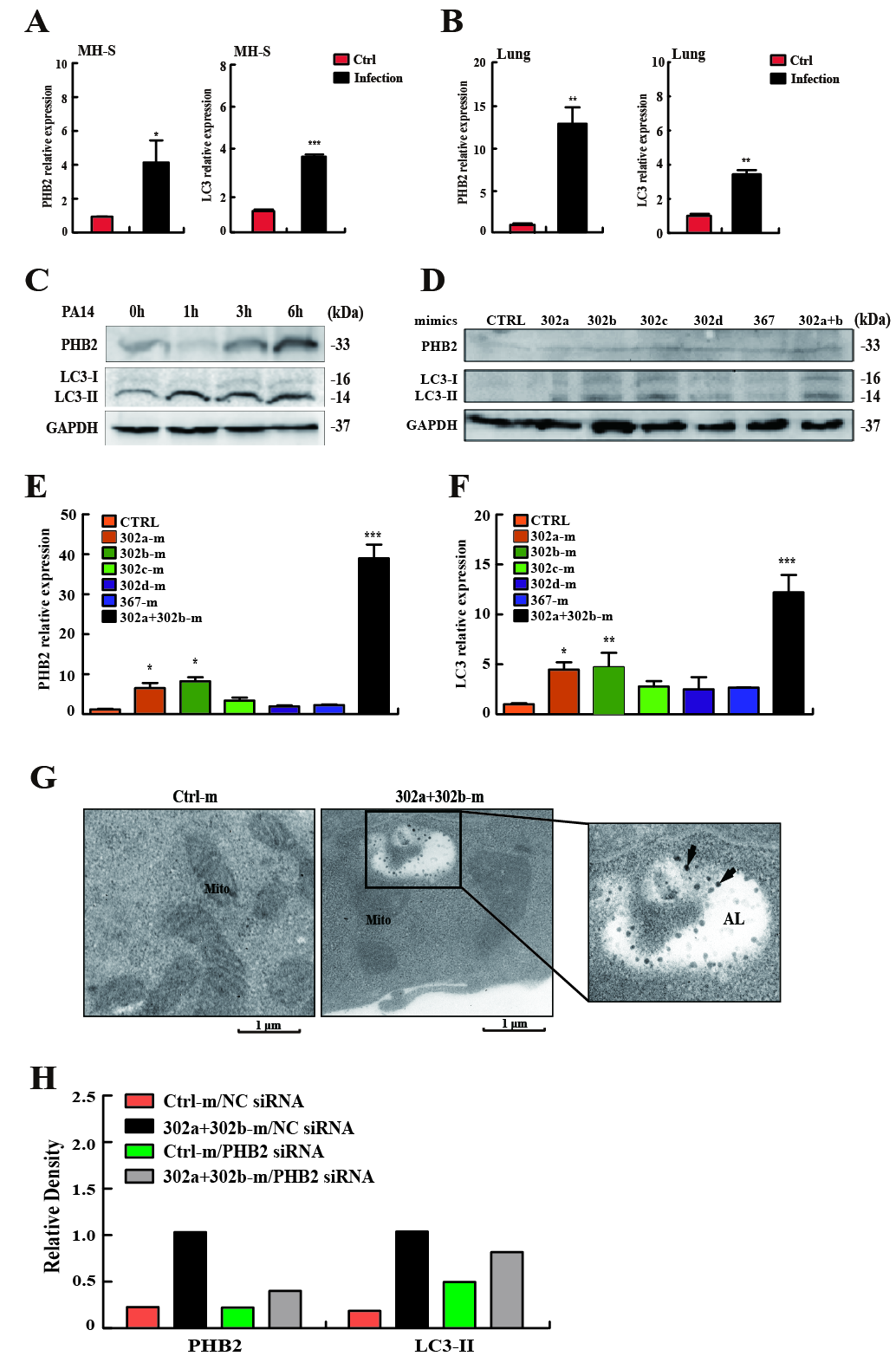

Supplement: Supplementary Figure 2 — P. aeruginosa infection and miR-302/367 cluster induces expression of mitophagy receptor in in vitro and in vivo. (A–C) MH-S cells were infected with PA14 at MOI 10:1 for 1 h and polymyxin B was added for another 1 h to kill bacteria outside of the cells (A and C). The cell samples were also collected at 6 h. Mice were treated with 1×10^7^ CFU of PA14 and the lung tissues samples (n = 3) were collected at 6 h (B). The expression level of PHB2 and LC3 in MH-S cells were detected by qPCR (A) and Western blotting (C). The expression level of PHB2 and LC3 in lung tissues samples were detected by qPCR (B). (D-F) The MH-S cells were transfected with each single miRNA of miR-302/367 cluster for 24 h. Expression levels of LC3 and PHB2 were detected by Western blotting (D) and qPCR (E and F). (G) Electron micrographs of MH-S cells stained with immunogold-conjugated anti-LC3 antibody to detect LC3. Cells were transfected with control or miR-302a and miR-302b mimics. Black arrows, immunogold particles labeling LC3. Mito, mitochondrion; AL, autolysosome. Scale bars, 1 μm. (H) Densitometric quantification of the Western blotting gel data from Figure 4A was used by Quantity one software and is shown as means. Data are shown as the mean ± SEM of three independent experiments. *P < 0.05; **, p < 0.01; ***, p < 0.001. [file Image_2.tif]

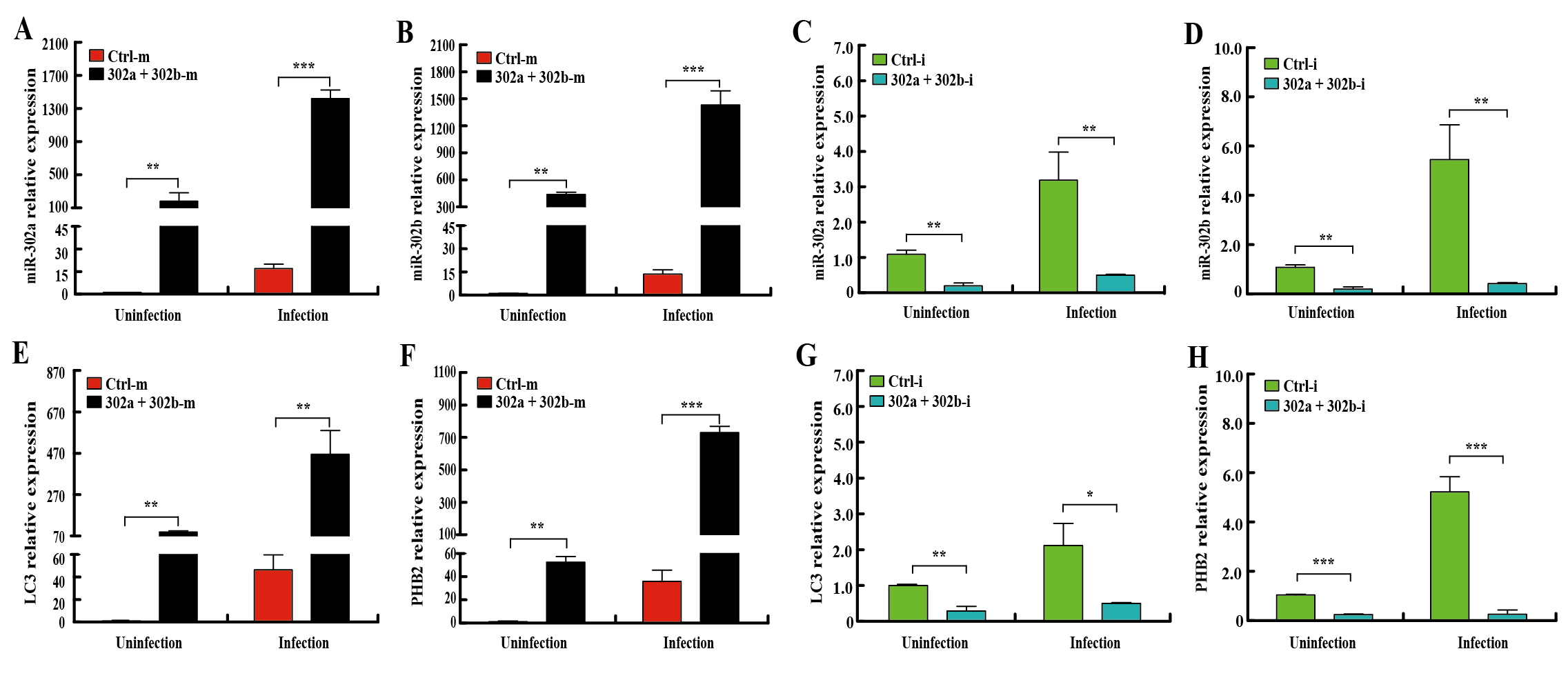

Supplement: Supplementary Figure 3 — miR-302/367 cluster induces expression of mitophagy receptor in human primary alveolar epithelial cells. (A–H) The human primary alveolar epithelial cells were transfected with miR-302a and miR-302b mimics (A, B, E, and F) or inhibitors (C, D, G, H) for 24 h and then either left uninfected or infected with PA14 for 6 h. Expression levels of miR-302a and miR-302b (A–D) or LC3 and PHB2 (E–H) were detected by qPCR. Data are shown as the mean ± SEM of three independent experiments. *P < 0.05; **, p < 0.01; ***, p < 0.001. [file Image_3.tif]
