# Supplementary material for: MicroRNA-302/367 Cluster Impacts Host Antimicrobial Defense via Regulation of Mitophagic Response Against Pseudomonas aeruginosa Infection
Source: Front Immunol. 2020 Oct 7;11:569173. doi: 10.3389/fimmu.2020.569173 (PMC7576609; doi:10.3389/fimmu.2020.569173)
Supplement: Supplementary file 4 [file Table_1.doc]

**Table S1. Primers of miRNAs, genes and luciferase reporter construct containing NF-κB 3' UTR**.

| ID | Primer Sequences (5'-3') |
| --- | --- |
| miR-302a-3p Sense | GCGGCGTAAGTGCTTCCATGTTTTG |
| miR-302b-3p Sense | GCGGCGTAAGTGCTTCCATGTTTTA |
| miR-302c-3p Sense | GCGGCGTAAGTGCTTCCATGTTTCA |
| miR-302d-3p Sense | GCGGCGTAAGTGCTTCCATGTTTGA |
| snoRNA202 Sense | GCCTTTTGAACCCTTTTCCATCTG |
| Mouse GAPDH Sense | ACCACAGTCCATGCCATCAC |
| Mouse GAPDH AntiSense | TCCACCACCTGTTGCTGT |
| Human GAPDH Sense | AGGGCTGCTTTTAACTCTGGT |
| Human GAPDH AntiSense | CCCCACTTGATTTTGGAGGGA |
| NF-κB Sense | ATGTAGTTGCCACGCACAGA |
| NF-κB AntiSense | GGGGACAGCGACACCTTTTA |
| Mouse LC3 Sense | CTTCGCCGACCGCTGTTA |
| Mouse LC3 AntiSense | ATCCGTCTTCATCCTTCTTCCTG |
| Human LC3 Sense | GAGAAGCAGCTTCCTGTTCTGG |
| Human LC3 AntiSense | GTGTCCGTTCACCAACAGGAAG |
| Mouse PHB2 Sense | CGGGCCAGACCTCGAAAAAT |
| Mouse PHB2 AntiSense | TTCGGATCAACAGGGACACC |
| Human PHB2 Sense | AAAATTGTGCAGGCCGAGGG |
| Human PHB2 AntiSense | TGGCTGGCTCCTCAAAAACT |
| PHB1 Sense | ATGGCTGCCAAAGTGTTTGAGTC |
| PHB1 Antisense | TCACTGGGGAAGCTGGAGAAGC |
| PINK1 Sense | GCTTGCCAATCCCTTCTATG |
| PINK1 Antisense | CTCTCGCTGGAGCAGTGAC |
| PARKIN Sense | CTGCGTGTGATTTTTGCC |
| PARKIN Antisense | GTGTCCAGAATGACCGCC |
| IL-1β Sense | GTCAACGTGTGGGGGATGAA |
| IL-1β Antisense | AAGCAATGTGCTGGTGCTTC |
| IL-6 Sense | CCCCAATTTCCAATGCTCTCC |
| IL-6 Antisense | CGCACTAGGTTTGCCGAGTA |
| TNF-α Sense | GGCAGGTTCTGTCCCTTTCA |
| TNF-α Antisense | CATCTTTTGGGGGAGTGCCT |
| FUNDC1 Sense | ATGGCATCCCGGAACCCCC |
| FUNDC1 AntiSense | AGATGCCAGGCCTAGCAAAAAG |
| NIX Sense | CCTCGTCTTCCATCCACAAT |
| NIX AntiSense | GTCCCTGCTGGTATGCATCT |
| BNIP3 Sense | GTTCCAGCCTCCGTCTCTAT |
| BNIP3 AntiSense | CTGTTGGTATCTTGTGGTGTCT |
| pGL3-Luc NF-κB Sense | CGGTCTAGATTTGGCGTCCTTTCTTGGTT |
| pGL3-Luc NF-κB AntiSense | CCGGATCCGGTCAACAATGTGCTTGTACTT |
| pGL3-Luc NF-κB Mutant Sense | CCGGATCCTTATAAAAGTAAAATAAAAAAGT |
